# Specific strains of *Streptococcus mutans*, a pathogen of dental caries, in the tonsils, are associated with IgA nephropathy

**DOI:** 10.1038/s41598-019-56679-2

**Published:** 2019-12-27

**Authors:** Seigo Ito, Taro Misaki, Shuhei Naka, Kaoruko Wato, Yasuyuki Nagasawa, Ryota Nomura, Masatoshi Otsugu, Michiyo Matsumoto-Nakano, Kazuhiko Nakano, Hiroo Kumagai, Naoki Oshima

**Affiliations:** 1 Department of Nephrology and Endocrinology, National Defense Medical College, Tokorozawa, Saitama, Japan; 2 Division of Nephrology, Seirei Hamamatsu General Hospital, Hamamatsu, Shizuoka, Japan; 3 Department of Nursing, Faculty of Nursing, Seirei Christopher University, Hamamatsu, Shizuoka, Japan; 4 Department of Pediatric Dentistry, Okayama University Graduate School of Medicine, Dentistry and Pharmaceutical Sciences, Okayama, Okayama, Japan; 5 Department of Pediatric Dentistry, Division of Oral infection and Disease Control, Osaka University Graduate School of Dentistry, Suita, Osaka, Japan; 6 Division of Kidney and Dialysis, Department of Internal Medicine, Hyogo College of Medicine, Nishinomiya, Hyogo, Japan

**Keywords:** Bacterial pathogenesis, Nephritis

## Abstract

*Streptococcus mutans* is known to be a major causative agent of dental caries, and strains expressing the cell surface collagen-binding Cnm protein contribute to the development of several systemic diseases. A relationship between tonsillar immunity and glomerulonephritis has been recognized in IgA nephropathy (IgAN), and specific pathogens may have effects on tonsillar immunity (mucosal immunity). Here, we present findings showing a relationship between the presence of Cnm-positive *S. mutans* strains in the tonsils of IgAN patients and IgAN condition/pathogenesis. Analyses of tonsillar specimens obtained from patients with IgAN (n = 61) and chronic tonsillitis (controls; n = 40) showed that the Cnm protein-positive rate was significantly higher in IgAN patients. Among IgAN patients, the tonsillar Cnm-positive group (n = 15) had a significantly higher proportion of patients with high urinary protein (>1.5 g/gCr) and lower serum albumin level than the Cnm-negative group (n = 46). Additionally, Cnm protein and CD68, a common human macrophage marker, were shown to be merged in the tonsils of IgAN patients. These findings suggest that Cnm-positive *S. mutans* strains in the tonsils may be associated with severe IgAN.

## Introduction

Patients with Immunoglobulin A nephropathy (IgAN), the most common cause of primary glomerulonephritis throughout the world^1,2^, have a poor renal prognosis^3^. Without undergoing specific therapy, more than one-third of these patients progress to end-stage kidney disease within 20 years^4,5^. Unfortunately, few disease-targeted treatment options are available because the pathogenesis of IgAN remains to be fully clarified^6^.

IgAN patients are often presented with macroscopic hematuria with upper respiratory infections, such as tonsillitis^7^. Hotta *et al*. were the first to proposed that a tonsillectomy along with steroid pulse therapy for IgAN patients would not only decrease hematuria/proteinuria but also result in improved remission rates^8^. The results of a recently published large-scale clinical study indicated that tonsillectomy is effective as treatment for IgAN^9^. Furthermore, clinical observations have indicated a relationship between tonsillar immunity and pathogenesis^10–12^. Tonsillar immunity is considered mucosal immunity: when exposed to pathogens (e.g. bacteria and their constituents), antigen-presenting cells such as macrophages become the starting point and cause B-cells and plasma cells to produce antibodies including IgA.

In comparisons of tonsils obtained from IgAN patients with those from tonsillar hyperplasia cases, the relative abundance of specific bacteria was found to be significantly different^13^, suggesting the tonsillar immune system or the hosts’ genetic make-up, other epigenetics or environmental factors behind the colonization of specific bacteria in the tonsils are important with respect to the pathogenesis of IgAN. Indeed, some bacteria, specifically periodontitis-related bacteria, have been referred to as the causal antigens or exacerbation factors in the pathogenesis of the disease^14–18^.

*Streptococcus mutans*, categorized as a Gram-positive facultative anaerobic oral streptococcal species, has been recognized as a major pathogenic agent in the development of dental caries in humans^19^. Furthermore, *S. mutans* has been reported to trigger infective endocarditis (IE) and has occasionally been isolated from blood specimens obtained from IE patients^20^. Some *S. mutans* strains express the cell surface collagen-binding protein Cnm, which is encoded by the *cnm* gene. Cnm shows an extracellular matrix-binding capability and can be a pathogenetic factor related to IE^21^. Previously, we reported that *S. mutans* strains showing Cnm expression were associated with aggravation of various systemic diseases, such as cerebral hemorrhage^22–24^, non-alcoholic steatohepatitis^25^, and inflammatory bowel disease^19^. Moreover, our additional studies showed a significantly higher carrier rate of Cnm-positive *S. mutans* strains in the saliva of IgAN patients than in that of non-IgAN controls, and Cnm-positive *S. mutans* strains present in the oral cavity may have effects on the severity of IgAN^26–28^.

The purpose of this study was to confirm the presence of Cnm-positive *S. mutans* in the tonsils, which was suggested to be more related to the severity of IgAN, and to investigate the correlation with clinical background.

## Results

### Generation of Cnm antiserum

**Figure 1:**
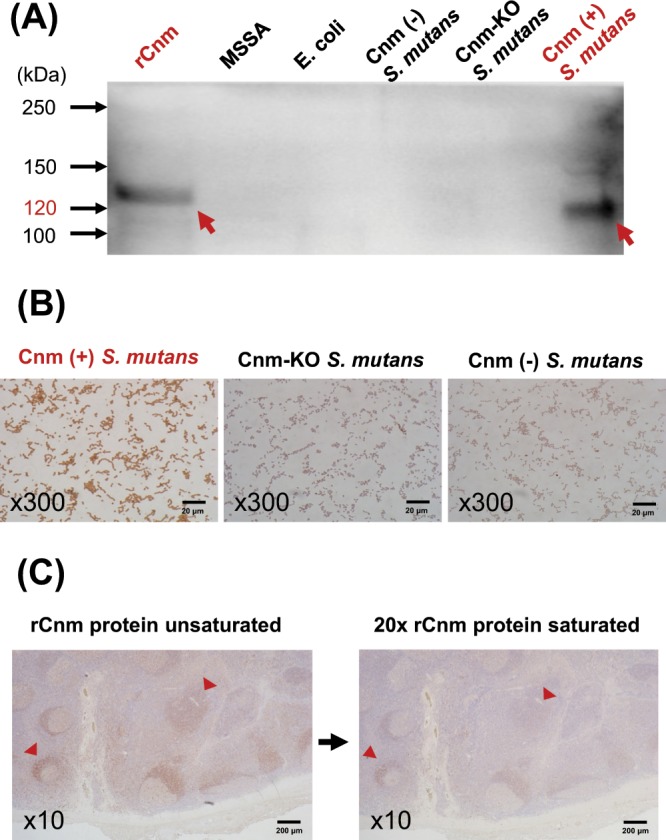
Construction of Cnm antiserum. (**A**) Western blotting of recombinant Cnm (rCnm) protein and cell-surface proteins of Cnm-negative strains: methicillin-susceptible *Staphylococcus aureus* (MSSA), *Escherichia coli*, Cnm (−) *S. mutans*, and Cnm-KO *S. mutans*, and the Cnm-positive strain Cnm (+) *S. mutans*. Arrows indicate positive bands at 120 kDa. (**B**) Culture smears with Cnm (+) *S. mutans*, Cnm-KO *S. mutans*, and Cnm (−) *S. mutans*. Immunohistochemistry findings showed that the Cnm-positive strain was stained and the Cnm-negative strains were not. (**C**) Serial sections of tonsils obtained from IgAN patient. Immunoperoxidase staining for Cnm, using Cnm antiserum unsaturated or saturated with rCnm protein, showed that saturating with rCnm protein diminished staining with Cnm antiserum (arrowheads).

To confirm the immunoreactive specificity of obtained antisera to Cnm-positive *S. mutans*, we used Cnm (+) *S. mutans*, Cnm (−) *S. mutans*, and Cnm-KO *S. mutans* cells. Methicillin-susceptible *Staphylococcus aureus* (MSSA) and *Escherichia coli* cells were used as negative controls. Western blot analysis demonstrated a positive band in Cnm (+) *S. mutans* whole cell extracts, whereas no bands were found in the MSSA, *E. coli*, Cnm (−) *S. mutans*, or Cnm-KO *S. mutans* extracts (Fig. 1A).

Immunohistochemistry findings of a cultured bacterial smears for Cnm showed a positive reaction for Cnm (+) *S. mutans* and a negative reaction for Cnm-KO *S. mutans* and Cnm (−) *S. mutans* (Fig. 1B). To confirm a specific reaction of Cnm antiserum to Cnm-positive *S. mutans* in the tonsil tissues, immunoperoxidase staining for Cnm was performed with serial tonsillar sections using Cnm antiserum either unsaturated or saturated with recombinant Cnm (rCnm) protein, which showed that saturation with rCnm protein diminished tonsillar staining by Cnm antiserum (Fig. 1C).

### Assessment of the degree of Cnm positivity in the tonsils from IgAN and chronic tonsillitis patients

**Figure 2:**
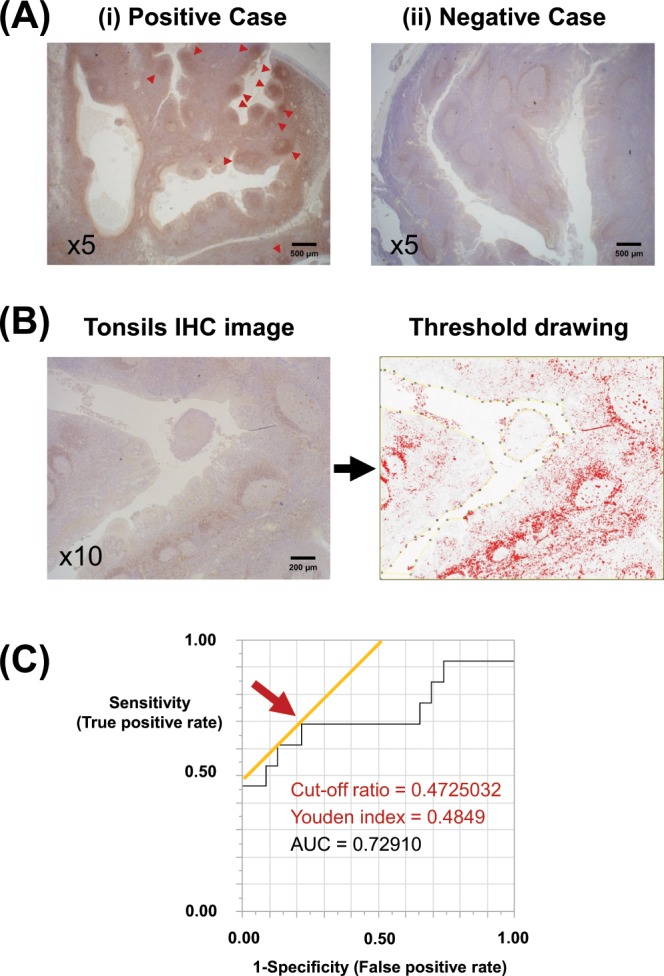
Assessment of degrees of Cnm-positive *S. mutans* in the tonsils from patients with IgAN and chronic tonsillitis. (**A**) Tonsillar sections were obtained from patients with IgAN and chronic tonsillitis. The degree of tonsillar immunoperoxidase staining for Cnm varied among the patients and disease group. (i) Representative tonsil from an IgAN patient (positive Case) showing strong staining, including germinal centers (arrowheads). (ii) Representative tonsil from an IgAN patient (negative Case) showing weak staining, even in the germinal centers. (**B**) Measurement of ratio of Cnm-positive area to the entire tonsil. Images taken at low magnification were converted to 8-bit red, green, and blue (RGB) images, and a threshold drawing was then generated using the image processing program, Fiji/ImageJ. (**C**) Receiver operating characteristic (ROC) curves for the ratio of Cnm-positive area to the entire tonsil for predicting *cnm* positivity in salivary specimens from IgAN patients by PCR. Arrow indicates the cut-off ratio yielding maximum sensitivity and specificity for the prediction of Cnm positivity in the specimen. The optimal cut-off ratio was 0.472 (n = 36, area under the curve = 0.72910; sensitivity = 61.5%; specificity = 87.0%).

The degree of immunoperoxidase staining for Cnm in tonsillar sections varied by patient and disease group (Fig. 2A). Figure 2A shows demonstrative findings from a strongly positive (i) and weakly negative case (ii). As for the positive case, the entire area including the germinal centers was well stained and the germinal centers were not stained, whereas, in the negative case, the mantle zone around them was only weakly stained. To evaluate the ratio of Cnm-positive area to the entire tonsil, images obtained with low magnification were converted to 8-bit red, green, and blue images, from which a threshold drawing was generated (Fig. 2B).

A receiver operating characteristic curve for identifying the cut-off ratio (Cnm-positive area to the entire tonsil) that provides optimal separation between the tonsillar Cnm-positive and Cnm-negative groups is shown in Fig. 2C. The optimal cut-off ratio for yielding maximum sensitivity and specificity for the prediction of *cnm* positivity in the saliva was 0.472 (n = 36, area under the curve = 0.72910; sensitivity 61.5%, specificity 87.0%).

### Characteristics of IgAN and chronic tonsillitis (control) groups

The interval between the kidney biopsy and tonsillectomy procedures was 11.2 ± 18.8 months (mean ± SD). The age of the IgAN group was significantly higher than that of the control group (*p* < 0.05). Additionally, the percentages of urinary protein excretion and urinary occult blood were significantly higher in the IgAN group (*p* < 0.0001). The estimated glomerular filtration rate (eGFR) tended to be lower in the IgAN than in the control group, although the difference was not significant (Table 1). The Cnm-positive area/total tonsillar area ratio was significantly higher in the IgAN group than the control group (*p* < 0.0001), as was the positive rate for Cnm (Cnm-positive patients/total patients) in the tonsils (24.5% vs. 0.0%, *p* = *0.0003*) (Table 1).

**Table 1:** Characteristics of IgAN and chronic tonsillitis (control) groups.

Years	IgAN group (n = 61)	Control group (n = 40)	*p-*value
Age (year; mean ± SD)	35.9 (32.9–38.9)	30.3 (27.4–33.2)	0.0359
Sex (M/F)	31/30	28/12	0.0655
Cnm-positive area/total tonsillar area ratio (mean ± SD)	0.32 ± 0.17	0.04 ± 0.06	<0.0001
Positive rate of Cnm-positive *S. mutans* in tonsils (%)	24.5	0.0	0.0003
Serum creatinine (mg/dL; mean ± SD)	0.8 ± 0.2	0.7 ± 0.1	0.4764
eGFR (mL/min; mean ± SD)	83.4 ± 19.5	90.9 ± 12.9	0.0658
% of urinary protein 1 + or higher	78.6	2.5	<0.0001
% of urinary occult blood 1 + or higher	95.0	4.7	<0.0001

SD: standard deviation, eGFR: estimated glomerular filtration rate

Bold values indicate statistical significance at *p* < *0.05*.

### Comparison between IgAN patients in Cnm-positive and Cnm-negative groups

For this comparison, we analyzed 61 IgAN patients (mean age, 35.9 ± 11.7 years; 31 males, 30 females). The percentage of those with urinary protein at 1.5 g/gCr or greater was higher in the Cnm-positive group (26.6% vs. 4.3%, *p* = *0.0280*), whereas serum albumin in that group was significantly lower (*p* = *0.0256*) (Table 2).

**Table 2:** Comparison between the Cnm-positive and Cnm-negative IgAN patients.

Characteristics	Cnm-positive (n = 15)	Cnm-negative (n = 46)	p-value
Age (year; mean ± SD)	39.0 (32.1–45.9)	34.9 (31.5–38.3)	0.2650
Sex (M/F)	9/6	22/24	0.5541
Duration from onset to treatment (year; mean ± SD)	5.2 ± 5.4	7.5 ± 9.4	0.9112
BMI (kg/m^2^; mean ± SD)	23.5 ± 5.9	21.3 ± 2.7	0.4754
Systolic blood pressure (mmHg; mean ± SD)	116.6 ± 17.1	117.8 ± 17.7	0.6817
Diastolic blood pressure (mmHg; mean ± SD)	70.0 ± 12.5	70.8 ± 13.4	0.7779
**Serum albumin (g/dL; mean ± SD)**	**3.8 ± 0.4**	**4.1 ± 0.3**	**0.0256**
Serum total cholesterol (mg/dL; mean ± SD)	216.7 ± 82.2	193.3 ± 28.9	0.5082
Serum LDL cholesterol (mg/dL; mean ± SD)	140.1 ± 83.7	111.9 ± 22.7	0.3461
Serum triglyceride (mg/dL; mean ± SD)	179.2 ± 167.7	104.8 ± 58.3	0.0789
Serum creatinine (mg/dL; mean ± SD)	0.8 ± 0.2	0.7 ± 0.1	0.1472
eGFR (mL/min/1.73 m^2^; mean ± SD)	75.5 ± 18.0	85.4 ± 19.5	0.1067
Serum IgA (mg/dL; mean ± SD)	350.1 ± 123.6	338.0 ± 117.9	0.4460
**% Urinary protein 1.5 g/gCr or higher**	**26.6**	**4.3**	**0.0280**
Urinary RBC score (0~3; mean ± SD)	2.1 ± 0.7	1.9 ± 0.8	0.4649

BMI: body mass index, eGFR: estimated glomerular filtration rate. SD: standard deviation,

RBC: red blood cell, LDL: low-density lipoprotein

Bold values indicate statistical significance at *p* < *0.05*.

There were no significant differences in age, sex, duration from onset to treatment (only tonsillectomy or combination of tonsillectomy and systemic steroid administration for approximately 1 year), body mass index, blood pressure (systolic, diastolic), serum total cholesterol, serum low-density lipoprotein cholesterol, serum triglyceride, serum creatinine, eGFR, serum IgA, and urinary red blood cell (RBC) score between the groups (Table 2).

### Analysis of kidney histology in IgAN patients

A total of 44 kidney biopsy samples from 61 IgAN patients (Cnm-positive, n = 10; Cnm-negative, n = 34) were analyzed, and histological findings were compared based on the Oxford classification. The segmental glomerulosclerosis and tubular atrophy/interstitial fibrosis scores in the Cnm-positive group tended to be higher (Table 3).

**Table 3:** Analysis of the kidney biopsy specimens of IgA nephropathy patients using the Oxford classification.

	Cnm-positive (n = 10)	Cnm-negative (n = 34)	*p-*value
Mesangial hypercellularity score	0.40	0.38	0.9207
Segmental glomerulosclerosis score	0.80	0.58	0.2264
Endocapillary hypercellularity score	0.50	0.38	0.5108
Tubular atrophy/interstitial fibrosis score	0.60	0.38	0.2272

### Cnm antiserum staining of glomeruli in IgAN patients

Cnm immunostaining of the kidney specimens was conducted on 119 cases, including 57 cases of IgAN patients in whom both tonsillar and kidney specimens were available, and 62 cases of IgAN patients, in whom only kidney specimens were available. Most of these IgAN patients did not show positive glomerular staining for Cnm, with only 4 suspected to have positive staining. However, we concluded that those were false positive findings.

### Relationship between galactose-deficient IgA1 glomerular deposition and tonsillar Cnm-positive rate in IgAN patients

**Figure 3:**
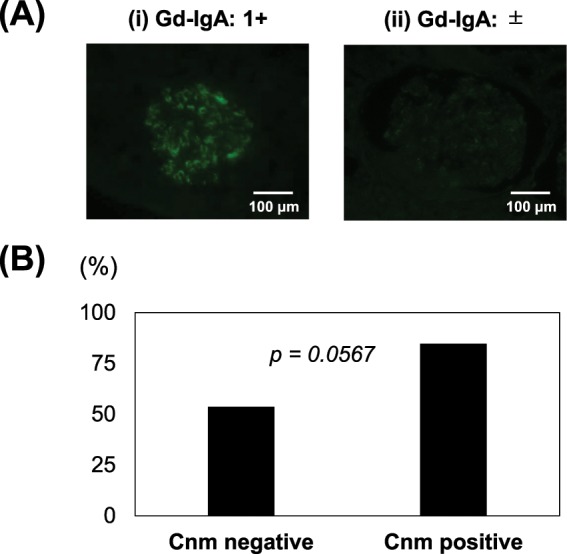
Relationship between glomerular deposition of galactose-deficient IgA1 (Gd-IgA1) and tonsillar Cnm-positive rate in IgAN patients. (**A**) IF staining for Gd-IgA1 in kidney sections from IgAN patients. Based on the degree of staining, IgAN patients were divided into two groups: (i) strong (1+) and (ii) weak (±). (**B**) The rate of glomerular Gd-IgA 1 + was 84.6% in the Cnm-positive group and 53.6% in Cnm-negative group (*p* = 0.0567).

Immunofluorescent (IF) staining for galactose-deficient IgA1 (Gd-IgA1) showed varying intensity (Fig. 3A). The rate of glomerular Gd-IgA 1 + was 84.6% in tonsils from the Cnm-positive group and 53.6% in those from the Cnm-negative group (*p* = *0.0567*) (Fig. 3B).

### Double IF staining for Cnm and CD68 in tonsillar sections from IgAN patients

**Figure 4:**
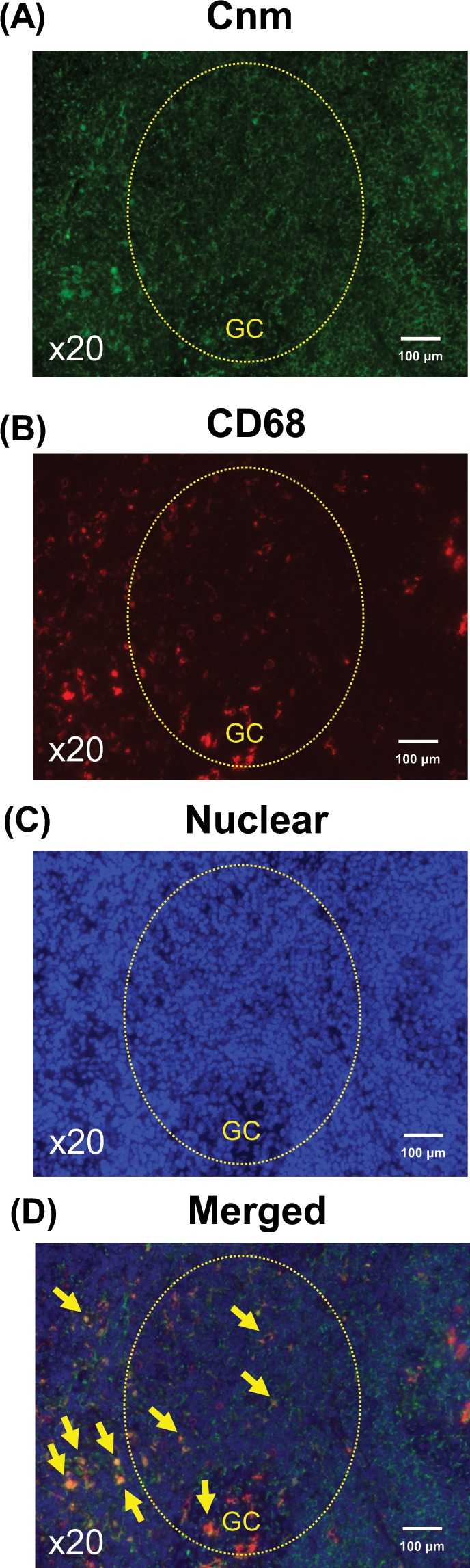
Double IF staining for Cnm and CD68 on tonsillar sections from IgAN patients. (**A–D**) Double IF staining for Cnm (Alexa Fluor 488: green) and CD68 (Alexa Fluor 594: red) and counterstaining of nuclei (DAPI: blue) were performed using tonsillar sections previously stained with immunoperoxidase for Cnm. (**A**) Cnm was predominantly observed in the germinal centers (GC) or surrounding area, with the surrounding area being more dominant. (**B**) CD68, a macrophage marker, was observed in the germinal centers and surrounding area. (**C**) Nuclear staining with DAPI. (**D**) Merged image of (**A–C**). Arrows indicate Cnm-positive macrophages (original magnification, A-D, × 20). Findings suggest that Cnm coexist with macrophages in the tonsils.

Double IF staining for Cnm (Alexa Fluor 488: green) and CD68, a macrophage marker (Alexa Fluor 594: red), was performed using tonsillar sections that had been stained with immunoperoxidase for Cnm. Cnm staining was observed mainly in the germinal center or the surrounding area. Figure 4A shows a representative Cnm-positive germinal center (yellow circle) at a high magnification. CD68 staining of the same area is shown in Fig. 4B, whereas DAPI is shown in Fig. 4C, with a merged image presented in Fig. 4D. Arrows indicate the colocalization of Cnm with CD68.

## Discussion

This is the first study to indicate that Cnm-positive *S. mutans* strains in the tonsils may be associated with severe IgAN. Our findings showed that the Cnm-positive area/total tonsillar area ratio was significantly higher in IgAN patients than in the control group (Table 1). Furthermore, Cnm protein was observed in the tonsils (Fig. 2A) but not in the kidneys of IgAN patients, whereas Cnm expression in the tonsils was associated with urinary protein level (Table 2). The degree of positivity for Gd-IgA1 in the kidneys tended to be higher in Cnm-positive patients than in Cnm-negative patients. Moreover, our results based on IF staining of the tonsils indicate the possibility of macrophage coexistence with Cnm-positive *S. mutans*. According to earlier reports, Cnm-positive rates in the saliva and areas affected by dental caries had significant correlations with urinary protein level in IgAN patients^26,27^. Nevertheless, those studies did not reveal a possible mechanism related to the involvement of Cnm-positive *S. mutans* in the development and severity of IgAN. Our findings suggest the association of Cnm-positive *S. mutans* with the severity of IgAN via their colonization in the tonsils.

The Cnm-positive group had a significantly higher proportion of patients with high urinary protein level and lower serum albumin level than the Cnm-negative group, while both tended to have a lower eGFR (Table 2). Proteinuria causes hypoalbuminemia, and both may sometimes coincide with the presence of IgAN^29^. Generally, urinary protein load in the renal tubules causes a decrease in eGFR via a decrease in post-glomerular flow^30^.

In IgAN patients, the predominant IgA subset in immune complexes deposited in the glomeruli was found to be IgA1, in which the O-glycan chains of the hinge region are underglycosylated (galactose-deficient IgA1)^31,32^. In IgAN patients, the most common extrarenal clinical manifestation is pharyngotonsillitis, which is strongly associated with exacerbation of hematuria and proteinuria^33,34^, phenomena that have been shown to be induced by the stimulation of the Gd-IgA1 production by infectious antigens in the tonsils^35^.

A rate of 1 + or higher for glomerular Gd-IgA1 tended to be more common in the present Cnm-positive group (Fig. 3B). Furthermore, double staining of Cnm and CD68 suggested that Cnm may coexist with macrophages in the tonsils (Fig. 4A–D). This seems to indicate the following two possibilities in relation to Cnm-positive *S. mutans* and tonsillar macrophages. First, tonsillar macrophage phagocytose Cnm-positive *S. mutans*. During the process of tonsillar immune response against foreign antigens, macrophages engulf the antigen and subsequently process and present it to CD4 + T-cells, which are then activated. Activation of CD4 + T-cells leads to the differentiation of antigen-specific B-cells, which finally differentiate into plasma cells and secrete Gd-IgA1^36,37^. Second, Cnm-positive *S. mutans* binds to the Fcγ receptors on macrophages by forming a complex with soluble CD89. It is known that CD89, an IgA receptor, is involved in the pathology of IgAN. The mechanism is that soluble CD89 forms a complex with Gd-IgA1 and binds to the Fc receptor γ-chain on immune cells such as macrophages to initiate inflammatory signaling^38^. Furthermore, *Streptococcus* can directly bind to CD89^39^. We have found the possibility of macrophage coexistence with Cnm in the tonsils. Few Cnm positive cells were detected in kidney biopsy tissues from our IgAN patients. Therefore, we suspect that the Cnm protein itself does not have direct effects on the kidneys.

This study has some limitations. First, in some of our IgAN patients, findings noting Cnm-positive *S. mutans* in tonsil examinations were not fully matched with the results of salivary testing. We employed immunohistochemical staining of the available paraffin-embedded tonsillar sections as the means for detecting the presence of Cnm in the tonsils. To determine the staining cut-off, we examined the correlation between Cnm positivity in the saliva and tonsils in 36 patients who had both tonsillar and salivary samples available because *cnm* detection by salivary PCR has already been established^40^. We found that the saliva was more likely to be positive for Cnm than the tonsils. We believe that there are several possible explanations for this result. The tonsils and saliva are in contact and the tonsils are in an anaerobic environment, whereas the saliva is in an aerobic one. *S. mutans* organisms prefer an aerobic environment. Additionally, we could not perform immunostaining for Gd-IgA1 in kidney sections from some IgAN patients because they had previously undergone a kidney biopsy, resulting in diagnosis at another hospital, and were subsequently referred to us for IgAN treatment, including tonsillectomy. Finally, it is possible that intraoral bacteria other than Cnm-positive *S. mutans* may be involved in the pathogenesis of IgAN. Indeed, we previously reported that superinfection with Cnm-positive *S. mutans* and *Campylobacter rectus* resulted in a significant increase in urinary protein among IgAN patients^28^.

In conclusion, the present results suggest that Cnm-positive *S. mutans* organisms harbored in the oral cavity are associate with IgAN severity via their colonization in the tonsils. This may support the significance of tonsillectomy as treatment for IgAN patients and the importance of oral care for the prevention of kidney disease. We propose a new concept of ‘oral-kidney association’, considering that *S. mutans* may affect the pathogenesis of IgAN. In order to further investigate the concept that intraoral infection can cause and aggravate IgAN, accumulation of additional clinical evidence is needed.

## Materials and Methods

### Subjects and clinical data

The subjects (n = 61) were treated for IgAN as outpatients at the National Defense Medical College Hospital, Tokorozawa, Japan and Seirei Hamamatsu General Hospital, Hamamatsu, Japan. Each had been previously diagnosed with IgAN based on kidney biopsy results, with histological diagnosis made based on light microscopy and IF findings. All patients were over 15 years of age and underwent tonsillectomy between 2006 and 2017 at the National Defense Medical College Hospital or Seirei Hamamatsu General Hospital^27^. For patients aged 15–19 years, the consent for our study protocol was obtained not only from them but also from their parents. Exclusion criteria were diagnosis of a secondary IgAN diseases (e.g. IgA vasculitis, lupus nephritis) and treatment with steroid or immunosuppression therapy. The control group (n = 40) was consisted of patients requiring tonsillectomy for chronic tonsillitis. At the time of the tonsillectomy, urinary protein excretion, urinary occult blood, urinary protein excretion/urinary creatinine excretion, urinary RBC count, serum creatinine, eGFR, serum IgA, serum albumin, serum total cholesterol, serum low-density lipoprotein cholesterol, serum triglyceride, blood pressure (systolic, diastolic), height, body weight, and body mass index were obtained. RBC score was defined as urinary RBC < 5/high power field (HPF): 0; 5–10/HPF:1; 10–30/HPF:2; and >30/HPF:3.

### Generation of antiserum against Cnm

The bacterial cells used in this analysis were from Cnm-positive and Cnm-negative strains. The Cnm-positive strains were SA31, TW295, and TW871 [referred to as Cnm (+) *S. mutans*]. Those were isolated from separate individual but were the same, as they each expressed Cnm protein. Cnm-negative strains included the standard strain MT8148 [referred to as Cnm (−) *S. mutans*] and its Cnm-defective isogenic mutant strains, including TW295CND [referred to as Cnm- knockout (KO) *S. mutans*], MSSA Terashima, and *E. coli* coliB.

We constructed TW295CND (Cnm-KO *S. mutans*) to be used as a negative control during western blotting and immunostaining with the Cnm antiserum. This mutant strain with insertional inactivation of the *cnm* gene was constructed as previously reported^41^. First, the *cnm* gene was amplified by PCR using TaKaRa Ex Taq (Takara Bio. Inc., Kusatsu) with primers constructed based on the *cnm* sequence from strain TW295 (GenBank accession no. AB469913). The amplified fragment was subsequently cloned into a pGEM-T Easy vector (Promega, Madison, WI) to generate pTN11. The middle of the open reading frame of *cnm* in pTN11 was cleaved by BsmI and was then ligated with the erythromycin resistance gene (*erm*) from the recombinant plasmid, which was linearized by digestion at the unique restriction site of PstI. Next, the plasmid was introduced into *S. mutans* TW295 using a homologous recombination method. The transformants were screened on MS agar plates containing erythromycin (10 mg/mL). The Cnm antiserum was generated by intramuscular administration of rCnm in rabbits, as previously described^42,43^. Briefly, antiserum against Cnm was generated by injecting rabbits (New Zealand white rabbits; Oriental Yeast Co. Ltd., Tokyo) with 4 intramuscular injections of purified rCnm (1200 μg) emulsified with a block copolymer adjuvant (Titer-Max Gold; CytRx Co., Atlanta, GA) every 2 weeks. The antibody titer of each antiserum sample was confirmed by enzyme-linked immunosorbent assay (ELISA) using rCnm. We collected blood from the rabbits prior to immunizing (day 0) and after the third injection of the rCnm/adjuvant (day 35). We then confirmed that the antibody titers were adequately elevated using ELISA. Specifically, after dilution of serums by 6400–25600 times, the absorbance ratios (day35/0) at an OD of 450 nm ranged from 22.6–28.3. To verify the specificity of the Cnm antiserum, western blotting of rCnm and whole bacterial cell extracts was performed.

Light microscopic immunohistochemistry was performed on smears with Cnm (+) *S. mutans*, Cnm (−) *S. mutans*, and Cnm-KO *S. mutans* using Cnm antiserum, with counterstaining performed with Mayer’s hematoxylin (Wako Chemicals, Osaka). As for the preparation of culture smear, we applied 5 μL of bacteria cultured in brain-heart Infusion medium (10 mL) to a slide glass with a sterile pipette and left it to stand for approximately 30 minutes to dry. Next, we dipped and fixed the slide glass in methanol for 2 minutes and then left the slide glass for approximately 30 minutes to dry.

The Ethics Committee of Animal Care and Experimentation, National Defense Medical College, Japan, approved all requests for animals and the intended procedures of the present study (permission no. 19044). All experiments were performed in accordance with relevant guidelines and regulations.

### Checking Cnm antiserum specificity

Fixing of tonsillar and kidney samples was performed with 10% formalin, and they were subsequently embedded in paraffin. For specificity verification, Cnm antiserum saturated with rCnm protein was used for immunoperoxidase staining of serial tonsillar sections.

### Assessment of Cnm positivity in tonsils of IgAN patients and chronic tonsillitis

For serial tonsillar sections, immunoreactivity against Cnm was quantitatively evaluated following two-step indirect immunoperoxidase staining for Cnm. Four images of each specimen were randomly obtained at low magnification and were subsequently analyzed using ImageJ-Fiji programs (ImageJ2, National Institutes of Health image)^44,45^ to determine the ratio of the area with Cnm positivity in the entire tonsil.

*S. mutans* strains were isolated from the saliva and confirmed using a previously described method^40^. Briefly, salivary specimens were collected in sterile phosphate-buffered saline and streaked onto mitis salivarius agar plates containing bacitracin and sucrose. We chose five colonies from each specimen and then extracted the genomic DNA from each strain. To confirm the identity of *S. mutans*, PCR was performed using *S. mutans*-specific primers sets (forward, 5′-GGC ACC ACA ACA TTG GGA AGC TCA GTT-3′; reverse, 50-GGA ATG GCC GCT AAG TCA ACA GGA T-3′), as previously described^46^. PCR was also performed to detect *cnm*-positive strains using *cnm*-specific primers sets (forward, 5′-GAC AAA GAA ATG AAA GAT GT-30; reverse, 50-GCA AAG ACT CTT GTC CCT GC-3′), as previously described^22^.

### Histological studies

The Oxford classification of IgAN includes four pathological variables that independently determine the risk for developing progressive renal disease—namely, mesangial hypercellularity (M0/M1 lesion), segmental glomerulosclerosis (S0/S1 lesion), endocapillary hypercellularity (E0/E1 lesion), and tubular atrophy/interstitial fibrosis (T0/T1/T2 lesion)^47,48^. We compared the renal histology of Cnm-positive and Cnm-negative groups of IgAN patients using the Oxford classification in a blind test.

### IF staining on tonsillar and kidney sections

We performed IF staining of renal biopsy specimens and tonsillar specimens obtained from patients who underwent tonsillectomy after having been diagnosed with IgAN and in whom both kidney and tonsillar specimens were available. Indirect double IF staining with Cnm antiserum and a mouse monoclonal antibody against human CD68, a marker for macrophages (PG-M1, Agilent), was performed using the following secondary antibodies: Alexa Fluor 488-labeled donkey anti-rabbit immunoglobulin G (IgG; Molecular Probes, Eugene, OR) and Alexa Fluor 594-labeled donkey anti-mouse IgG (Molecular Probes).

IF staining for Gd-IgA1 in kidney tissues from IgAN patients was performed using KM55, a Gd-IgA1- specific monoclonal antibody that has specific immunoreactivity for glomerular IgA deposition and can thus reflect the pathogenesis of IgAN^49^. Gd-IgA1 staining has been reported to be useful for diagnosis of IgAN^50^ and is hence performed with biopsy samples at many institutions, including ours, for such diagnosis. The Alexa Fluor 488-labeled donkey anti-rabbit IgG (Molecular Probes) secondary antibody was used for detection. Based on the degree of staining, IgAN patients were divided into two groups: (i) strong (1+) and (ii) weak (±) (there was no patient of negative (−) staining). We calculated the percentage of the number of patients in (i) to the number of ones in the IgAN group.

### Ethics

This study was conducted in full adherence to the Declaration of Helsinki (64th WMA General Assembly, Fortaleza, Brazil, 2013), and the study protocol was approved by the Ethics Committee of National Defense Medical College (approval no. 2690) and Seirei Hamamatsu General Hospital (approval no. 1807). All subjects were informed about the protocol and provided written consent prior to their participation.

### Statistics

All quantitative values are expressed as means ± standard deviation. When a significant difference was found, further statistical analysis of the difference between groups was performed using Fisher’s PLSD test, Mann–Whitney U test, or Fisher’s exact test, with *p* < *0.05* considered to indicate statistical significance. All statistical analyses were performed using the JMP software package (version 14; SAS Institute Inc., Cary, NC).
